# Circadian clock regulation of cone to horizontal cell synaptic transfer in the goldfish retina

**DOI:** 10.1371/journal.pone.0218818

**Published:** 2019-08-28

**Authors:** Christophe Ribelayga, Stuart C. Mangel

**Affiliations:** 1 Department of Neuroscience, College of Medicine, The Ohio State University, Columbus, Ohio, United States of America; 2 Department of Ophthalmology and Visual Science, McGovern Medical School, The University of Texas Health Science Center at Houston, Houston, Texas, United States of America; 3 MD Anderson/UTHealth Graduate School of Biomedical Sciences, The University of Texas Health Science Center at Houston, Houston, Texas, United States of America; Doheny Eye Institute/UCLA, UNITED STATES

## Abstract

Although it is well established that the vertebrate retina contains endogenous circadian clocks that regulate retinal physiology and function during day and night, the processes that the clocks affect and the means by which the clocks control these processes remain unresolved. We previously demonstrated that a circadian clock in the goldfish retina regulates rod-cone electrical coupling so that coupling is weak during the day and robust at night. The increase in rod-cone coupling at night introduces rod signals into cones so that the light responses of both cones and cone horizontal cells, which are post-synaptic to cones, become dominated by rod input. By comparing the light responses of cones, cone horizontal cells and rod horizontal cells, which are post-synaptic to rods, under dark-adapted conditions during day and night, we determined whether the daily changes in the strength of rod-cone coupling could account entirely for rhythmic changes in the light response properties of cones and cone horizontal cells. We report that although some aspects of the day/night changes in cone and cone horizontal cell light responses, such as response threshold and spectral tuning, are consistent with modulation of rod-cone coupling, other properties cannot be solely explained by this phenomenon. Specifically, we found that at night compared to the day the time course of spectrally-isolated cone photoresponses was slower, cone-to-cone horizontal cell synaptic transfer was highly non-linear and of lower gain, and the delay in cone-to-cone horizontal cell synaptic transmission was longer. However, under bright light-adapted conditions in both day and night, cone-to-cone horizontal cell synaptic transfer was linear and of high gain, and no additional delay was observed at the cone-to-cone horizontal cell synapse. These findings suggest that in addition to controlling rod-cone coupling, retinal clocks shape the light responses of cone horizontal cells by modulating cone-to-cone horizontal cell synaptic transmission.

## Introduction

The vertebrate retina is able to detect and transmit visual images in a moonless night, in the midday sun, and at all times in between when ambient light intensity varies by more than 10 orders of magnitude [1,2]. This remarkable ability relies on the presence of two different sets of photoreceptor, namely rods and cones, and on the control of retinal processing by light/dark adaptation mechanisms and endogenous circadian clocks [1–8]. Circadian clocks are self-autonomous oscillators that persist with a period of ~24 hours in the absence of light [9]. In the retina, circadian clocks orchestrate many aspects of retinal physiology and function on a daily basis, including photoreceptor disc shedding, synthesis of dopamine and the neurohormone melatonin, transcriptional activity, and visual sensitivity [3–8]. However, despite this large body of research, the physiological processes that the retinal clocks affect during day and night and the means by which the clocks control these processes remain unresolved.

**Fig 1:**
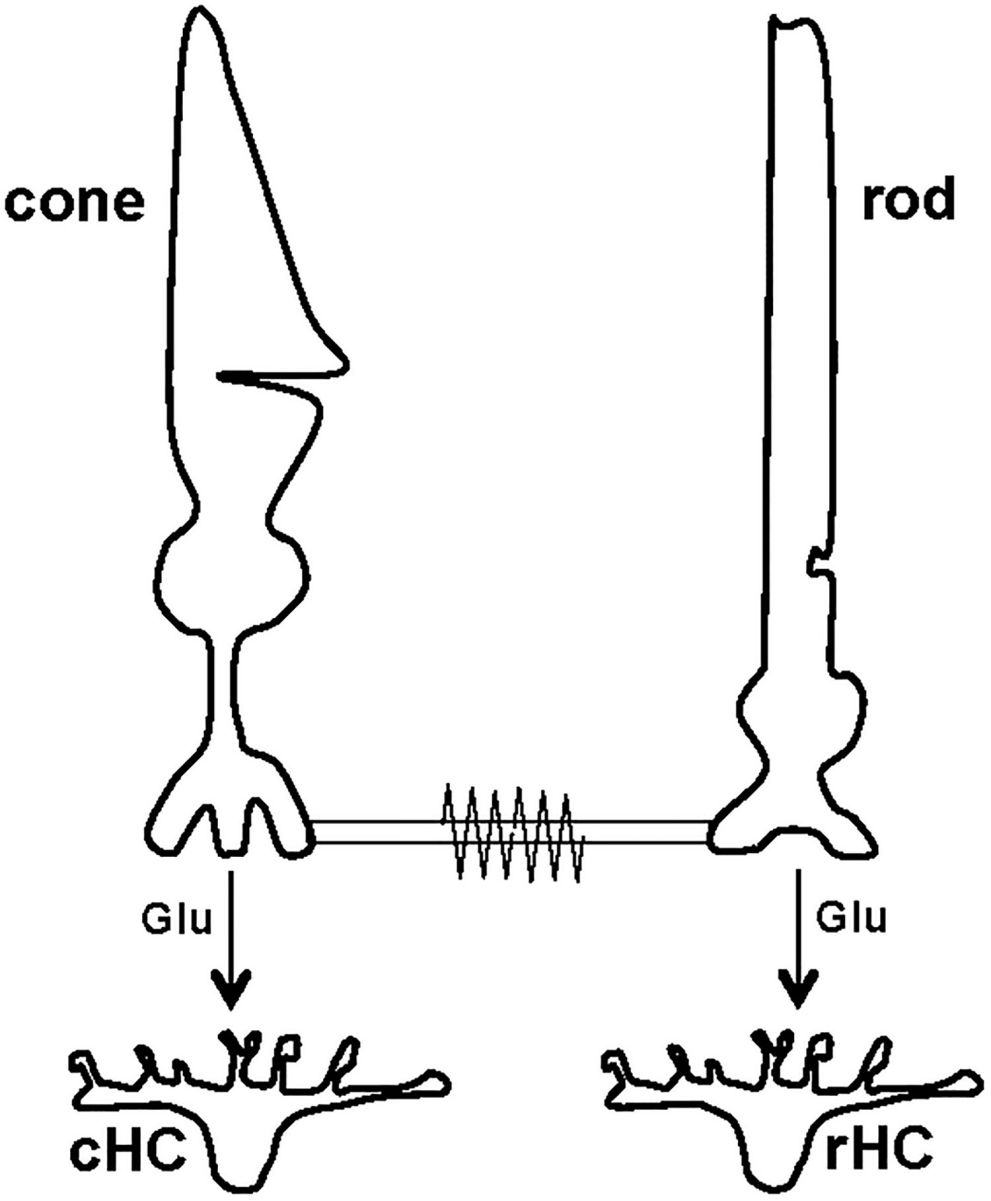
Schematic diagram showing signaling between cone and rod photoreceptor cells and synaptically-connected second order neurons in the goldfish retina. Signaling between a cone and a cone horizontal cell (cHC) and between a rod and a rod horizontal cell (rHC) is shown. Cones and rods use glutamate (Glu) to signal cHCs and rHCs, respectively. Also shown is a gap junction between a cone pedicle and rod spherule that is open at night in the dark and closed in the day.

Due to their different inherent sensitivity to light, isolated rods absorb very dim light (scotopic; see Methods for definitions of light levels) and produce photoresponses, whereas isolated cones require 2- to 3-fold brighter light to initiate light responses [1,2]. In the intact retina, both cones and rods are synaptically connected to second-order neurons, namely, cone horizontal cells (cHCs) and rod horizontal cells (rHCs), respectively (see Fig 1). In addition, in all vertebrate species, rod signals can enter cone circuits before the cone chemical synapse, i.e., through tiny intercellular channels or gap junctions that mediate electrical signaling between rod spherules and cone pedicles, the cell compartments from which transmitter is released to rHCs and cHCs, respectively (Fig 1) [10–18]. The physiological consequence of open rod-cone gap junctions is that the cone photoresponse not only reflects changes in the *intrinsic* cone photoresponse (i.e. photocurrent triggered by the light-activated phototransduction cascade and voltage-dependent conductances of cones), but also the influence of the photocurrent from neighboring rods that flows into the cones through gap junctions [12,14–16]. The resultant cone photovoltage controls the opening probability of the voltage-gated calcium channels at the cone pedicle and thereby synaptic transmission between cones and second-order neurons such as bipolar and horizontal cells [1,2].

We previously demonstrated that a circadian clock in the goldfish retina controls the activity of rod and cone pathways by controlling the conductance of rod-cone gap junctions [19]. Specifically, the clock modulates rod-cone coupling so that coupling is minimal during the day and remarkably robust at night [19]. The circadian variations in the extent of rod-cone coupling result in an increased convergence of rod signals to cones and cHCs at night [19–22]. Here, we set out to assess the extent to which circadian modulation of the light responses of cones and cHCs reflects changes in the degree of rod-cone coupling, cone-to-cHC synaptic mechanisms, and/or the intrinsic cone photoresponse. We compared the light response kinetics of cones, cHCs and rHCs and analyzed the cone-to-cHC transfer function during day and night under different illumination conditions. Our analysis reveals that in addition to controlling rod-cone coupling, retinal clocks likely control cone-to-cHC synaptic activity.

## Results

This study is based on analyses of the light response properties of cones, cHCs and rHCs in freshly isolated, intact goldfish neural retinas maintained by superfusion. Because we were not able to record from rods due to their small size, we used data collected from rHCs as an index of the rod photoresponse. Because previous studies demonstrated that similar day/night differences in the light responses of dark-adapted cones [19], cHCs [20,21] and rHCs [20,23] were observed under both circadian conditions (i.e. prolonged dark adaptation > 12 h) and during a regular light/dark cycle (i.e. dark adaptation > 1 h), the dark-adapted data were pooled into 2 groups: day-dark-adapted (i.e. day and subjective day data) and night-dark-adapted (night and subjective night data). Similarly, data collected under bright-light adapted conditions (i.e. photopic light adaptation > 1 h) during day or subjective day and night or subjective night were organized into two groups: day-light-adapted and night-light-adapted, respectively.

### The retinal clock controls the light responses of goldfish cones and cHCs but not rHCs

**Fig 2:**
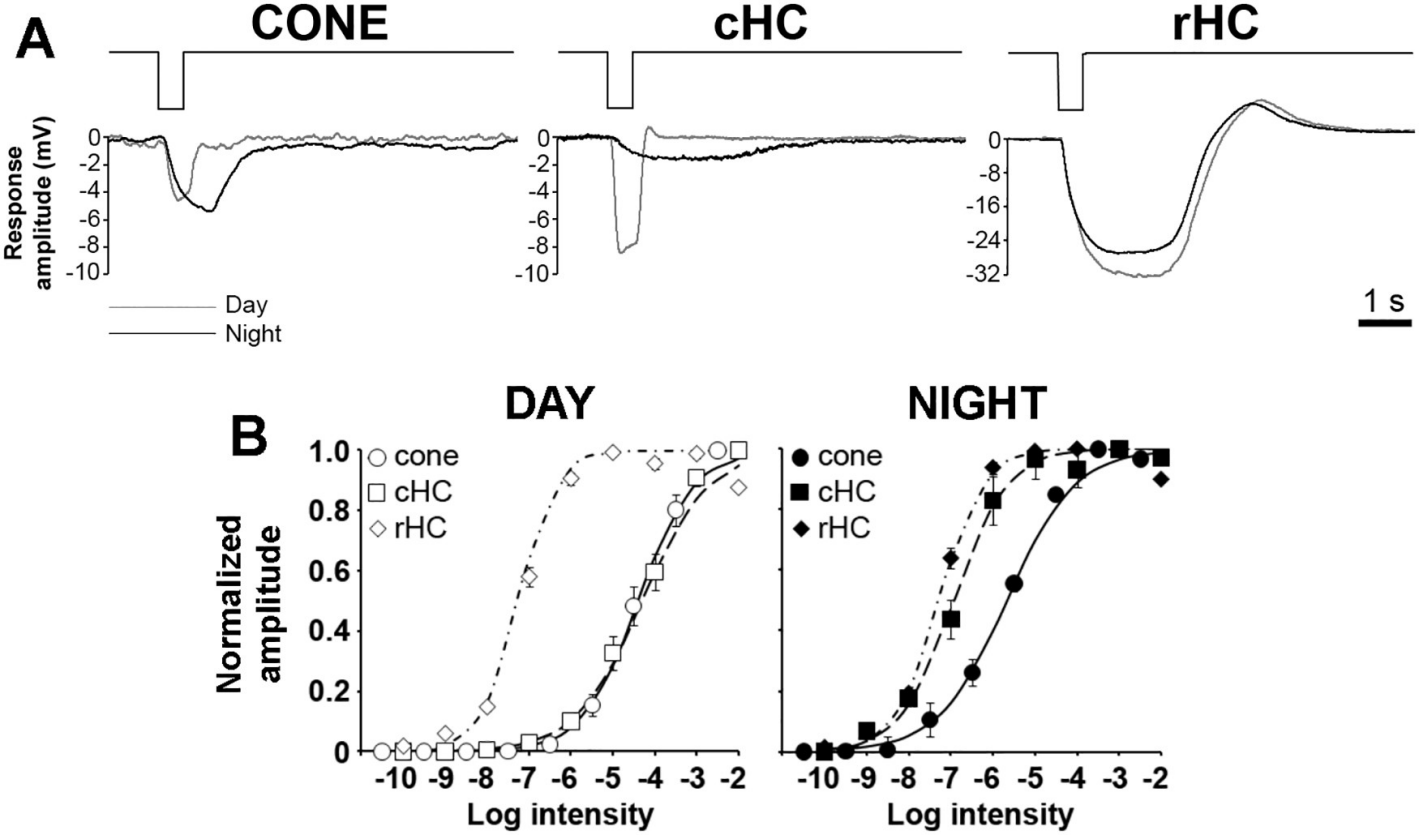
Light responses of dark-adapted cones, cHCs and rHCs during day and night. (A) Representative examples of dark-adapted cone (left panel), cHC (middle panel) and rHC (right panel) responses to a full-field white light stimulus (500 ms; -5 log *I*_o_) recorded during the day (grey traces) and night (black traces). (B) Normalized intensity-response functions of dark-adapted cones (circles), cHCs (squares) and rHCs (diamonds) during the day (open symbols; left panel) and night (filled symbols; right panel).

The light responses of cones, cHCs and rHCs to a 500 ms full-field white light stimulus were compared between day and night under dark-adapted conditions. Fig 2A shows typical examples of the responses to light stimuli in the low mesopic range (-5 Log *I*_o_) during day and night. Clear day/night differences were observed for cone and cHC responses. The light responses recorded during the day were similar to classic responses reported for these cells [24,25], but the nighttime responses were slower and longer in duration. The slow repolarization at light-offset is reminiscent of a rod-mediated “tail” or “plateau potential” [2,20]. Note that at this intensity, the cone response was larger at night compared to the day whereas the cHC response was smaller at night compared to the day and to the cone response at night (Fig 2A). In contrast, no obvious difference was found between the daytime and nighttime responses of rHCs. These data are consistent with increased rod input to cones and cHCs at night and agree well with published data [19–23].

The peak amplitude of the light responses was measured at different intensities and the normalized intensity-response curves were fit to a Hill-type function (Fig 2B; see Methods). The response thresholds (5% of maximum normalized amplitude) of cones and cHCs were decreased by ~ 2 log units at night, consistent with increased rod input at night. Comparison of the curves revealed that during the day, the light responses of cHCs roughly followed that of cones and could be clearly distinguished from that of rHCs in terms of both sensitivity and operating range (Fig 2B; Table 1). At night, the intensity-response function of the 3 cell types shared a high degree of similarity (Fig 2B; Table 1). The increased sensitivity of cones and cHCs at night is consistent with an increase in rod-cone coupling and rod input to cones and cHCs at night [19–21]. The rHC intensity-response function did not change between day and night, which indicates that the light responses of rHCs may not be controlled by a retinal clock, in agreement with previous reports [20,23].

**Table 1:** Light response kinetics of goldfish cones, cHCs and rHCs under dark-adapted conditions in the day and night.

	cell	RMP	V_max_	*n/r*^*2*^	*σ*	*N*	*n/r*^*2*^
**Day**	cone	-32 ± 1	15.6 ± 8	16/0.84	-4.32 ± 1.13	0.62 ± 0.07	16/0.91
	cHC	-35 ± 1	18.4 ± 1.2	7/0.79	-4.71 ± 0.96	0.62 ± 0.06	7/0.96
	rHC	-33 ± 1	19.9 ± 1.2	14/0.67	-7.17 ± 0.59	0.87 ± 0.06	14/0.98
**Night**	cone	-33 ± 1	6.4 ± 0.6^a^	10/0.57	-6.10 ± 1.98^a^	0.51 ± 0.07^a^	10/0.89
	cHC	-36 ± 1	1.8 ± 0.1^a^	7/0.67	-6.71 ± 2.35^a^	0.49 ± 0.07^a^	7/0.89
	rHC	-34 ± 1	17.0 ± 2.4	19/0.58	-7.28 ± 0.68	0.83 ± 0.05	19/0.97

Experimental data are averages ± SEM and residues from non-linear analysis are averages ± SD. RMP: resting membrane potential (mV); V_max_: maximum response amplitude (mV); *n*: sample size; *r*^*2*^: correlation coefficient; *σ*: semi-saturating constant (i.e. intensity to generate half-maximum amplitude; log *I*_*o*_)); *N*: Hill coefficient;

^a^: *P* < 0.05 compared to day value (Student’s t-test).

We reasoned that if the light responses of cones and cHCs at night only reflected the increased rod input to cones due to increased rod-cone electrical coupling at night [19], the response kinetics of cones and cHCs would closely match those of rHCs. Alternatively, retinal clocks may control other aspects of cone and cHC light responses, such as the intrinsic cone photoresponse and/or cone-to-cHC synaptic transmission. These additional actions of the clock would impact the response characteristics of cones and/or cHCs.

**Fig 3:**
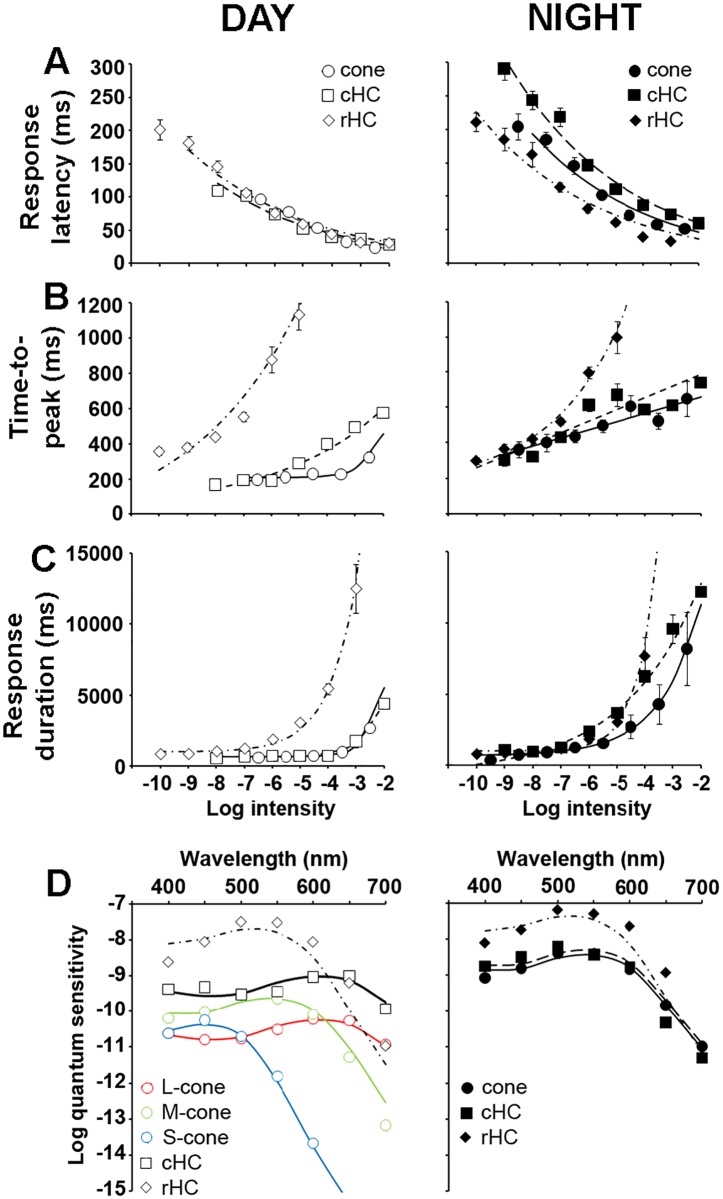
Light response kinetics and spectral sensitivity of dark-adapted cones, cHCs and rHCs in the day and night. (A-C) Normalized response latency (A), time-to-peak (B) and response duration (C) of dark-adapted cones (circles), cHCs (squares) and rHCs (diamonds) during the day (open symbols; left panel) and night (filled symbols; right panel). The responses of only 1 cell per retina to the full series of light stimuli were recorded because stimuli of intensities > -4.5 log *I*_*o*_ light-adapted the cells. Note the increased response latency and response duration of cones and cHCs at night. (D) Spectral sensitivity of cones, cHCs and rHCs recorded under dark-adapted conditions during the day (left) and night (right). During the day, the spectral sensitivity of dark-adapted cones fit one of three spectral sensitivity curves corresponding to the three major known cone subtypes in goldfish: L (~ 608 nm, red cone nomogram), M (~ 539 nm, green cone nomogram), and S (~ 451 nm, blue cone nomogram). The spectral sensitivity of all cones (filled circles), cHCs (filled squares) and rHCs (filled diamonds) recorded at night peaked at ~ 525 nm. The spectral sensitivity of rHCs at night closely fit a rod nomogram (dashed curve). The spectral sensitivity of cones and cHCs fit a template that combined the rod nomogram and that of an L-cone visual pigment (dotted curve) [19].

To determine whether this is the case, we compared the light response kinetics of cones, cHCs and rHCs during day and night under dark-adapted conditions (Fig 3A, 3B and 3C). Although the nighttime increase in time-to-peak (Fig 3B) and response duration (Fig 3C) were consistent with increased rod input to cones and cHCs, we found that response latency in cones and cHCs, but not rHCs, were dramatically affected by the clock at night (Fig 3A). Specifically, the response latency of cones and cHCs were longer at night than in the day but the response latency of rHCs was the same in day and night. Moreover, rHC response latency was shorter at night than that of cones and cHCs, and cHC response latency was 10 to 50 ms longer than that of cones (Fig 3A). Although sluggish cone light responses at night likely reflect increased rod input to cones, the even slower cHC response latency at night suggests additional clock-controlled mechanisms at the level of the cone-to-cHC synapse.

The spectral sensitivity of cones, cHCs and rHCs was measured during day and night (Fig 3D; Table 2). During the day, cones could be sorted in 3 different subtypes based on their spectral sensitivity: L (~ 608 nm, red cones), M (~ 539 nm, green cones), and S (~ 451 nm, blue cones). L-type (H1) cHCs showed peak sensitivity at ~ 610 nm consistent with major L-cone synaptic input to these cells [26]. rHC spectral sensitivity peaked at ~ 530 nm (Fig 3D; Table 1). These values are consistent with a large body of literature on the spectral properties of goldfish cones and HCs [25–28] and with our own spectral sensitivity measurements during the day [19–23]. In contrast, the spectral sensitivity of all cones and cHCs recorded at night matched that of rHCs (Fig 3D; Table 2), consistent with increased rod input at night that shifts the spectral sensitivity peak toward that of the rod pigment [19,20,29].

**Table 2:** Spectral characteristics of goldfish cones, cHCs and rHCs under dark-adapted conditions in the day and night.

	cell	RS	λ_max_	*n/r*^*2*^	*k*	*n/r*^*2*^	MIR
**Day**	L-cone	0.13 ± 0.17	613 ± 3	9/0.60	-10.30 ± 0.05	9/0.33	
	M-cone	3.30 ± 0.11	525 ± 2	6/0.95	-9.62 ± 0.16	6/0.66	3.72
	S-cone	ND	451 ± 2	1/0.99	-10.37 ± 0.06	1/0.99	
	cHC	0.28 ± 0.23	600 ± 5	8/0.36	-9.02 ± 0.05	8/0.30	
	rHC	4.46 ± 0.10	524 ± 3	5/0.89	-7.58 ± 0.16	5/0.76	0.34
**Night**	cone	2.97 ± 0.15^b^	535 ± 3^b^	10/0.84	-8.44 ± 0.08^b^	10/0.69	2.46
	cHC	3.58 ± 0.52^a^	527 ± 3^a^	4/0.92	-8.26 ± 0.10^a^	4/0.87	1.62
	rHC	3.97 ± 0.25	521 ± 3	5/0.91	-7.35 ± 0.16	5/0.81	0.20

Experimental data are averages ± SEM and residues from non-linear analysis are averages ± SD. RS: relative sensitivity (log(S_500_/S_700_)); λ_max_: peak of sensitivity (nm); *n*: sample size; *r*^*2*^: correlation coefficient; *k*: absolute sensitivity at the peak; MIR: mean isomerization rate per rod (Rh*.rod^-1^.s^-1^) calculated from *k* for λ_max_ ~ 500 nm (see Methods);

^a^: *P* < 0.05 compared to day value;

^b^: *P* < 0.05 compared to day L-cone value (Student’s t-test).

ND: not determined.

**Fig 4:**
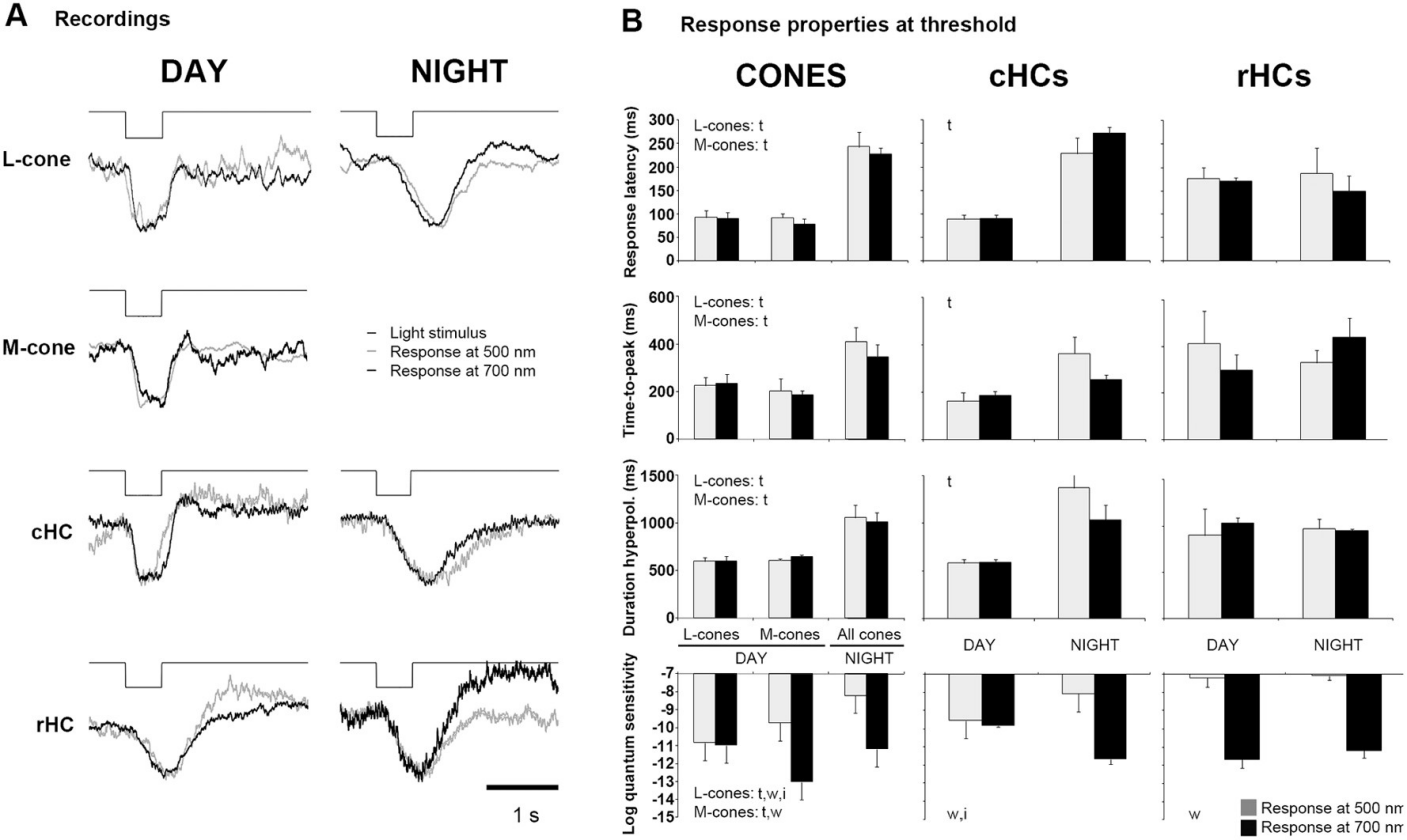
Threshold responses of dark-adapted cones, cHCs and rHCs to 500 nm and 700 nm light stimuli during day and night. (A) Representative examples of dark-adapted cone, cHC and rHC threshold responses (0.5 mV) to a light stimulus (500 ms) at 500 nm (grey trace) and 700 nm (black trace) during the subjective day (left column) and night (right column). The amplitude of each trace has been normalized for better comparison. (B) Averaged response latency, time-to-peak, duration of the hyperpolarization, and quantum sensitivity measured in cones (left column), cHCs (center column) and rHCs (right column) under the conditions described in (A). Data in (B) were analyzed with a two-WAY ANOVA, the first factor was time of day (t) and the second factor was wavelength (w). Significant variations (α = 0.05) are indicated on the left side of each figure; i: w x t interaction.

Another way to estimate rod input to cones and cHCs is to measure the relative sensitivity of the cells to 500 nm compared to 700 nm stimuli [15,30] (see Methods). Relative sensitivity (log (S_500_/S_700_)) was ∼ 0.2 for L-cones and cHCs during the day but was ∼ 3.0 for M-cones during the day and for all cones and cHCs at night (Table 2). The nighttime shift in relative sensitivity of cones and cHCs likely reflected substantial rod input to cones at night because the relative sensitivity of rHCs, which reflects that of rods, was ∼ 4.3 (Table 2). Although an increased contribution of M-cones at night cannot be excluded, it was likely minimal because M-cone light response threshold was more than 1 log *I*_o_ above that of cones at night (Table 2). In addition, the possibility that we sampled only M-cones at night is very unlikely based on the fact that cones at night were as sensitive to green light as L-cones during the day (Figs 3D and 4) and that our sampling was biased towards the bigger cones, which in goldfish are L-cones [31]. Together with the similarity of the time course of the light responses of cones, cHCs and rHCs, the spectral sensitivity data are consistent with an increase in rod-cone coupling and increased rod input to cones that dominates their light responses and subsequently those of cone-connected second-order neurons at night.

### The cone photoresponse is altered by the time of day

To further investigate the day/night differences in the light responses of dark-adapted cones, we compared the responses of cones at threshold (0.5 mV) to a 500 ms-full field light stimulus at 500 nm and 700 nm (± 10 nm). Because rod-cone coupling is weak during the day, we reasoned that the daytime responses of cones would display the characteristic fast and transient cone-like (“pure” cone) responses at both 500 nm and 700 nm, although with different sensitivities. In contrast, at night, due to the increase in rod-cone coupling and a peak of sensitivity at ~ 500 nm that is driven by rods, cone light responses would exhibit all of the characteristics of a rod response (i.e. slow time course) at 500 nm. However, cone light responses at night would exhibit a mixture of cone and rod characteristics (i.e. transient component and slow recovery) at 700 nm because at threshold, cones are as sensitive to red light as rods at night (Fig 3D).

Fig 4A shows that during the day both L- and M-cones responded to 500 nm and 700 nm light stimuli with characteristic “pure” cone kinetics. However, the time-course of the cone responses was much slower at night (Fig 4). At 500 nm, this observation was expected and consistent with a rod-driven cone response. At 700 nm, however, cone responses were very similar to those at 500 nm and lacked the characteristic initial fast and transient components. This result indicates that the light responses of cones at night at threshold are either entirely driven by rods or that intrinsic cone response kinetics are slower compared to the day and match those of rods. The nighttime responses of cHCs to both light stimuli also showed increased latency at threshold (Fig 4). In contrast, the time-course of rHC responses at threshold were invariant of wavelength and time-of-day, although a strong difference in quantum sensitivity was observed in the responses to 500 and 700 nm light stimuli (Fig 4B).

### Cone-to-cHC synaptic transfer is under circadian control

**Fig 5:**
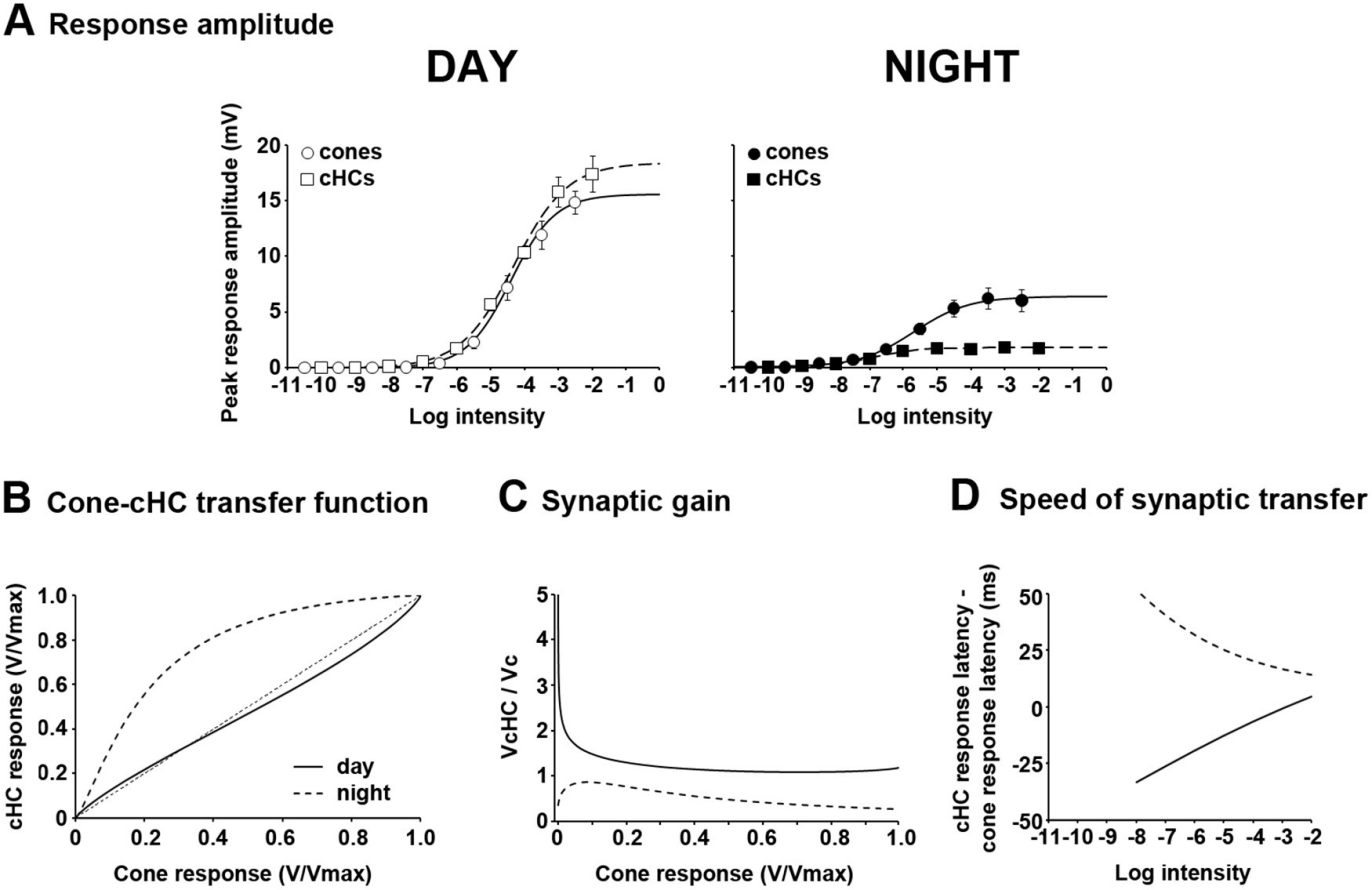
Characteristics of the cone-to-cHC synaptic transfer function during day and night under dark-adapted conditions. (A) Intensity-response functions of dark-adapted cones (circles) and cHCs (squares) during the day (open symbols; left panel) and night (filled symbols; right panel). Data points were fit to an allometric-type function (see Methods). The responses of only 1 cell per retina to the full series of light stimuli were recorded. Note that the maximum response amplitude (V_max_) is much lower at night for both cones and cHCs. (B) The cone-cHC transfer function was calculated from the normalized intensity-response functions illustrated in Fig 2B. The deviation of the transfer function from the identity line suggests that signal transmission from cones to cHCs is non-linear. Note the increased non-linearity of the function at night. (C) Synaptic gain calculated from the curves illustrated in (A). Note the high gain for low intensities during the subjective day and the low gain (< 1) at all intensities at night. (D) Speed of synaptic transfer was calculated from the curves shown in Fig 3A. Note that synaptic transfer is significantly longer at night compared to the day.

In order to further characterize cone-to-cHC synaptic behavior, we calculated the transfer function, the synaptic gain, and the speed of synaptic transfer during day and night under dark-adapted conditions (see Methods). Fig 5A illustrates averaged data of the responses of dark-adapted cones and cHCs to full-field white light stimuli during day and night. During the day, cone and cHC intensity-response functions followed a similar trend. Both saturated at about the same intensity (∼ -2 log *I*_o_) and exhibited similar maximum amplitudes (V_max_) ∼ 18–20 mV (Fig 5A, Table 1). In contrast, at night the two curves saturated at different light intensities and showed a much lower V_max_ compared to the day. In addition, cHCs exhibited a lower V_max_ than cones (Fig 5A, Table 1). The transfer function of the cone-to-cHC synapse was generated from the normalized peak amplitude intensity-response functions of cHCs (shown in Fig 2B) and cones (Fig 2B; see Methods). The linearity of the transfer function was tested against the identity line (Fig 5B) and by calculating the voltage gain of the system (V_cHC_/V_c_) as a function of normalized cone responses (Fig 5C). During the day, the cone-to-cHC transfer function was slightly non-linear, had its highest and lowest gains for dim and bright light stimuli, respectively, and exhibited an asymptotic synaptic gain value ∼ 1.5 (Fig 5C). In contrast, at night the cone-to-cHC transfer function was highly non-linear (Fig 5B) and revealed saturation of cHC responses at relatively low intensity stimuli (Fig 5A). Although the highest gain at night was observed for the dimmest stimuli, the gain decreased toward an asymptotic value ∼ 0.3, roughly 5 times lower than the lowest gain value in the day (Fig 5C). These observations are in agreement with previous reports on the non-linearity of the synaptic transfer function between cones and cHCs under dark-adapted conditions in poikilotherms [32–34]. Moreover, the results demonstrate that cone-to-cHC synaptic transmission is under circadian clock control so that it is quasi-linear in the day but highly non-linear at night with decreased gain.

The speed of synaptic transfer was calculated by plotting the difference between the response latency of cones and cHCs as a function of stimulus intensity (Fig 5D). During the day the latency of cHC light responses was shorter than that of cone light responses (see Fig 3A). Thus, the difference in the latencies was always negative irrespective of stimulus intensity (Fig 5D). In addition, the latency difference was larger for dim stimuli and smaller for brighter ones with an asymptotic value ~ 0 (Fig 5D). Overall, these observations are consistent with the large amount of convergence of cones onto cHCs (~ 35 to 1) [26] and the high synaptic gain at low light intensities (Fig 5B). In contrast, at night the latency of cone light responses was shorter than that of cHC light responses. Thus, the synaptic delay at night was always positive with a maximum value for dim light stimuli and a minimum value for bright light stimuli (Fig 5D). Therefore, the combination of high convergence of cones onto cHCs and low synaptic gain at night resulted in delayed cHC light responses at night. In summary, cone-to-cHC synaptic transmission is significantly longer at night compared to the day. Moreover, the increased non-linearity of the cone-cHC transfer function at night and the changes in synaptic gain and in the speed of synaptic transfer indicate that the retinal clock controls cone-to-cHC synaptic transmission so that at night the synapse is sensitive to small changes in the intensity of dim light stimuli and suppressed in its response to changes in brighter intensities.

### Bright light adaptation abolishes circadian control of cones and cHCs light responses

**Fig 6:**
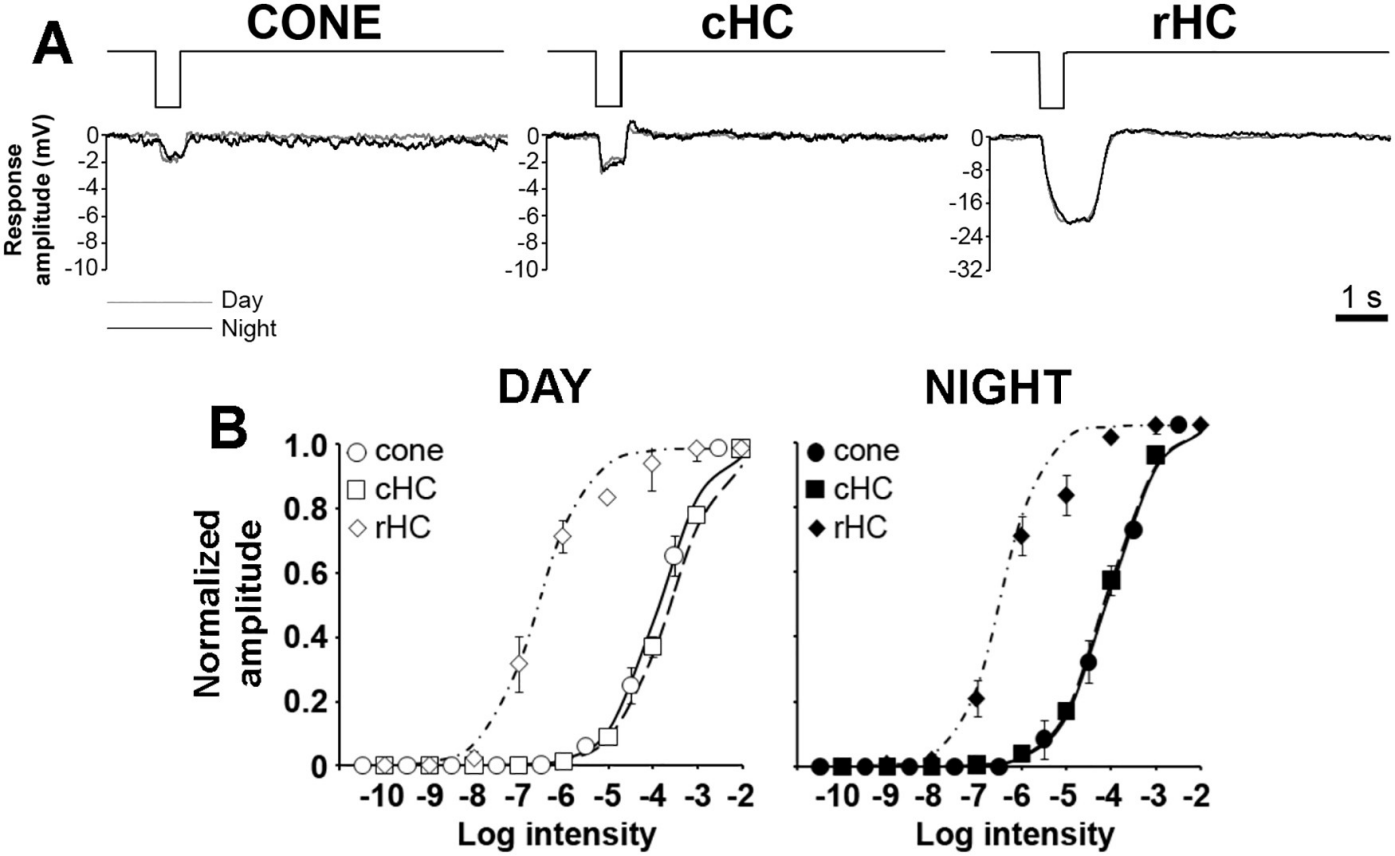
Light responses of bright light-adapted cones, cHCs and rHCs in the day and night. (A) Representative examples of bright (photopic) light-adapted cone (left panel), cHC (middle panel) and rHC (right panel) responses to a full-field white light stimulus (500 ms; -5 log *I*_o_) recorded during the day (grey traces) and night (black traces). (B) Normalized intensity-response functions of light-adapted cones (circles), cHCs (squares) and rHCs (diamonds) during the day (open symbols; left panel) and night (filled symbols; right panel).

Light adaptation is known to dynamically control gap junction permeability [17,18] and alter the effects of retinal clocks on retinal function [4–7]. The light response properties of cones, cHCs and rHCs were altered under bright (photopic) light-adapted conditions in the day and night. Fig 6A illustrates examples of responses of each of the 3 cell types to the same intensity stimulus (-5 log *I*_o_) during day and night under bright light-adapted conditions. No clear difference was evident between the daytime and nighttime recordings in each case. In addition, both the daytime and nighttime recordings resembled those obtained during the day under dark-adapted conditions with respect to the time course of the responses (see Fig 2). The sensitivity however was lower under light-adapted conditions compared to day-dark-adapted, i.e., the intensity-response curves following light adaptation were shifted to the right by ∼ 0.5 log *I*_o_ (compare Fig 6B with Fig 2B, and σ values in Table 3 with Table 1). In addition, the nighttime responses of cones and cHCs under light-adapted conditions lacked the rod component present at night under dark-adapted conditions (compare Fig 6A with Fig 2A), suggesting a suppressive effect of bright light adaptation on rod-cone coupling and rod input to cones and cHCs, as has been reported for cones [19] and cHCs [20].

**Table 3:** Light response kinetics of goldfish cones, cHCs and rHCs under light-adapted conditions in the day and night.

	cell	RMP	V_max_	*n/r*^*2*^	*σ*	*N*	*n/r*^*2*^
**Day**	cone	-33 ± 1	11.2 ± 1.0	11/0.79	-3.77 ± 1.02	0.70 ± 0.09	11/0.93
	cHC	-36 ± 1	25.1 ± 1.9	22/0.74	-3.65 ± 0.33	0.77 ± 0.04	22/0.98
	rHC	-34 ± 1	4.4 ± 0.3	5/0.79	-6.59 ± 1.35	0.80 ± 0.11	5/0.92
**Night**	cone	-38 ± 1^a^	11.0 ± 1.0	10/0.77	-3.90 ± 1.09	0.66 ± 0.08	10/0.94
	cHC	-34 ± 1	16.8 ± 0.8^a^	14/0.85	-4.09 ± 0.42^a^	0.81 ± 0.06	14/0.97
	rHC	-36 ± 1	18.7 ± 1.3^a^	6/0.86	-6.57 ± 1.09	1.08 ± 0.14^a^	6/0.96

Experimental data are averages ± SEM and residues from non-linear analysis are averages ± SD. RMP: resting membrane potential (mV); V_max_: maximum response amplitude (mV); *n*: sample size; *r*^*2*^: correlation coefficient; *σ*: semi-saturating constant (i.e. intensity to generate half-maximum amplitude; log *I*_*o*_)); *N*: Hill coefficient;

^a^: *P* < 0.05 compared to day value (Student’s t-test).

**Fig 7:**
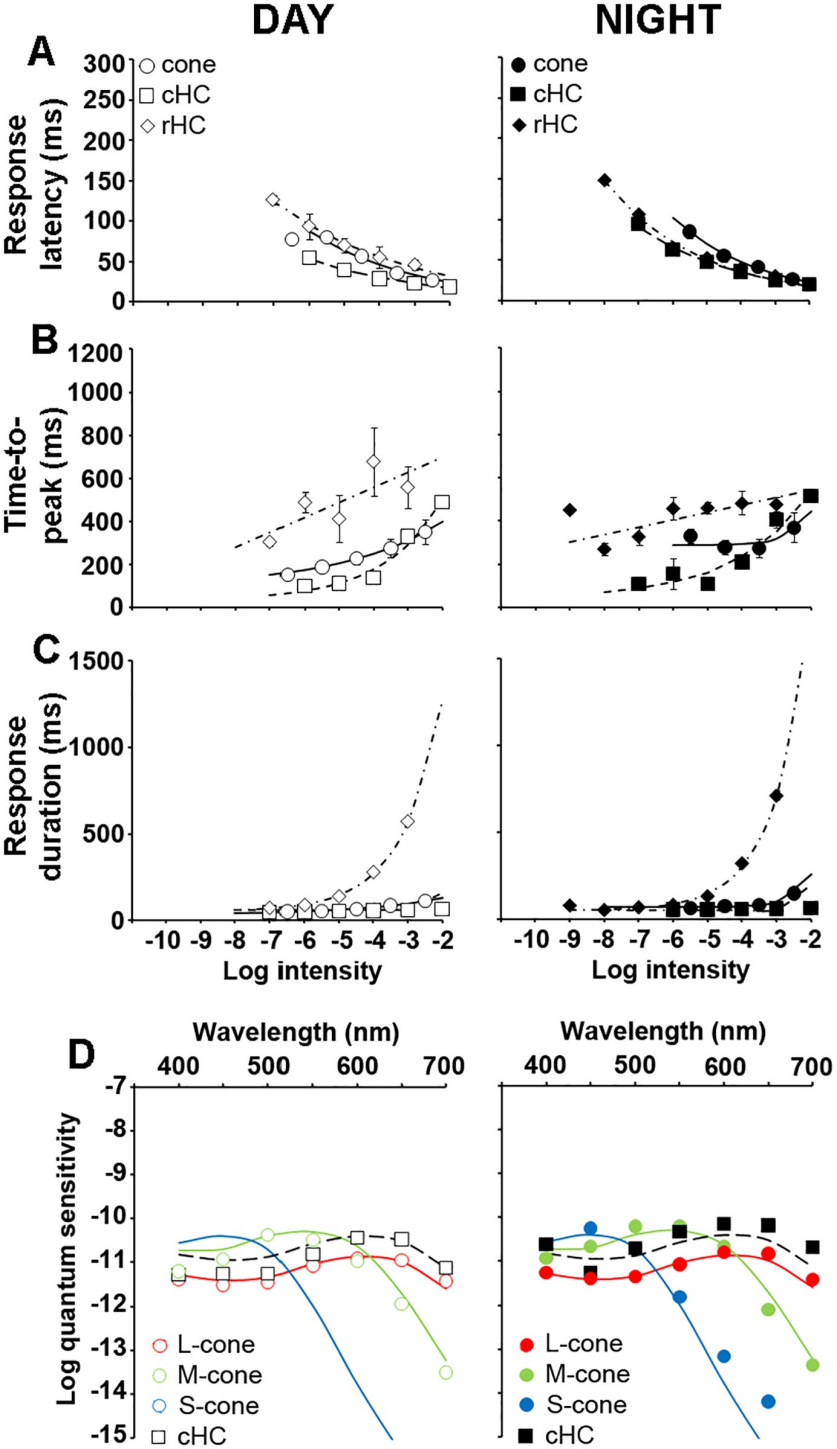
Light response kinetics and spectral sensitivity of bright light-adapted cones, cHCs and rHCs in the day and night. (A-C) Normalized response latency (A), time-to-peak (B) and response duration (C) of bright (photopic) light-adapted cones (circles), cHCs (squares) and rHCs (diamonds) during the day (open symbols; left panel) and night (filled symbols; right panel). Note that for each cell type, the daytime and nighttime intensity-response curves are similar and resemble those recorded during the day under dark-adapted conditions. (D) Spectral sensitivity of cones, cHCs and rHCs recorded under bright light-adapted conditions during the day (left) and night (right). Both during the day and night the spectral sensitivity of light-adapted cones fit one of three spectral sensitivity curves corresponding to the three major known cone subtypes in goldfish: L (~ 608 nm, red cone nomogram), M (~ 539 nm, green cone nomogram), and S (~ 451 nm, blue cone nomogram). The spectral sensitivity of cHCs (squares) matched that of L-cones in both day and night. We did not record any S-cones during the day and did not determine the spectral sensitivity properties of rHCs under light-adapted conditions.

Comparison of the light response kinetics, and analysis of the synaptic transfer function and the speed of signaling under light-adapted conditions revealed clear differences with that which was observed under dark-adapted conditions. Indeed, none of the response kinetics following light adaptation was different between day and night (Fig 7A, 7B and 7C) and they all typically resembled those recorded during the day under dark-adapted conditions (Fig 3A, 3B and 3C). However, the response latencies of cones and cHCs were relatively shorter during both day and night following light adaptation (Fig 7A) compared to nighttime under dark-adapted conditions (Fig 3A). The spectral sensitivity data were also consistent with bright light-induced suppression of rod-cone coupling [19]. Specifically, cones could be sorted into 3 different subtypes based on their spectral sensitivity (i.e. L, M and S) and L-type (H1) cHCs were most sensitive to red light regardless of the time of day (Fig 7D; Table 4). The absence of day/night differences in the light responses of cones, cHCs and rHCs and the suppressive effect of bright light adaptation on rod input to cones and cHCs agree well with our previous observations [19–23].

**Table 4:** Spectral characteristics of goldfish cones, cHCs and rHCs under light-adapted conditions in the day and night.

	cell	RS	λ_max_	*n/r*^*2*^	*k*	*n/r*^*2*^	MIR
**Day**	L-cone	-0.04 ± 0.02	619 ± 3	2/0.96	-10.91 ± 0.08	2/0.47	
	M-cone	3.13 ± 0.25	533 ± 3	6/0.90	-10.52 ± 0.09	6/0.83	295
	S-cone	ND	ND	ND	ND	ND	
	cHC	-0.36 ± 0.21	614 ± 8	9/0.18	-10.62 ± 0.10	9/0.15	
	rHC	ND	ND	ND	ND	ND	ND
**Night**	L-cone	0.07 ± 0.13	615 ± 3	4/0.77	-10.82 ± 0.05	4/0.45	
	M-cone	3.16 ± 0.20	527 ± 3	5/0.92	-10.27 ± 0.10^a^	5/0.83	166
	S-cone	ND	463 ± 5	1/0.96	-10.41 ± 0.20	1/0.96	
	cHC	1.94 ± 0.56^a^	616 ± 13	1/0.63	-10.23 ± 0.09	1/0.68	
	rHC	ND	ND	ND	ND	ND	ND

Experimental data are averages ± SEM and residues from non-linear analysis are averages ± SD. RS: relative sensitivity (log(S_500_/S_700_)); λ_max_: peak of sensitivity (nm); *n*: sample size; *r*^*2*^: correlation coefficient; *k*: absolute sensitivity at the peak; MIR: mean isomerization rate per rod (Rh*.rod^-1^.s^-1^) calculated from *k* for λ_max_ ~ 500 nm (see Methods);

^a^: *P* < 0.05 compared to day value (Student’s t-test).

ND: not determined.

**Fig 8:**
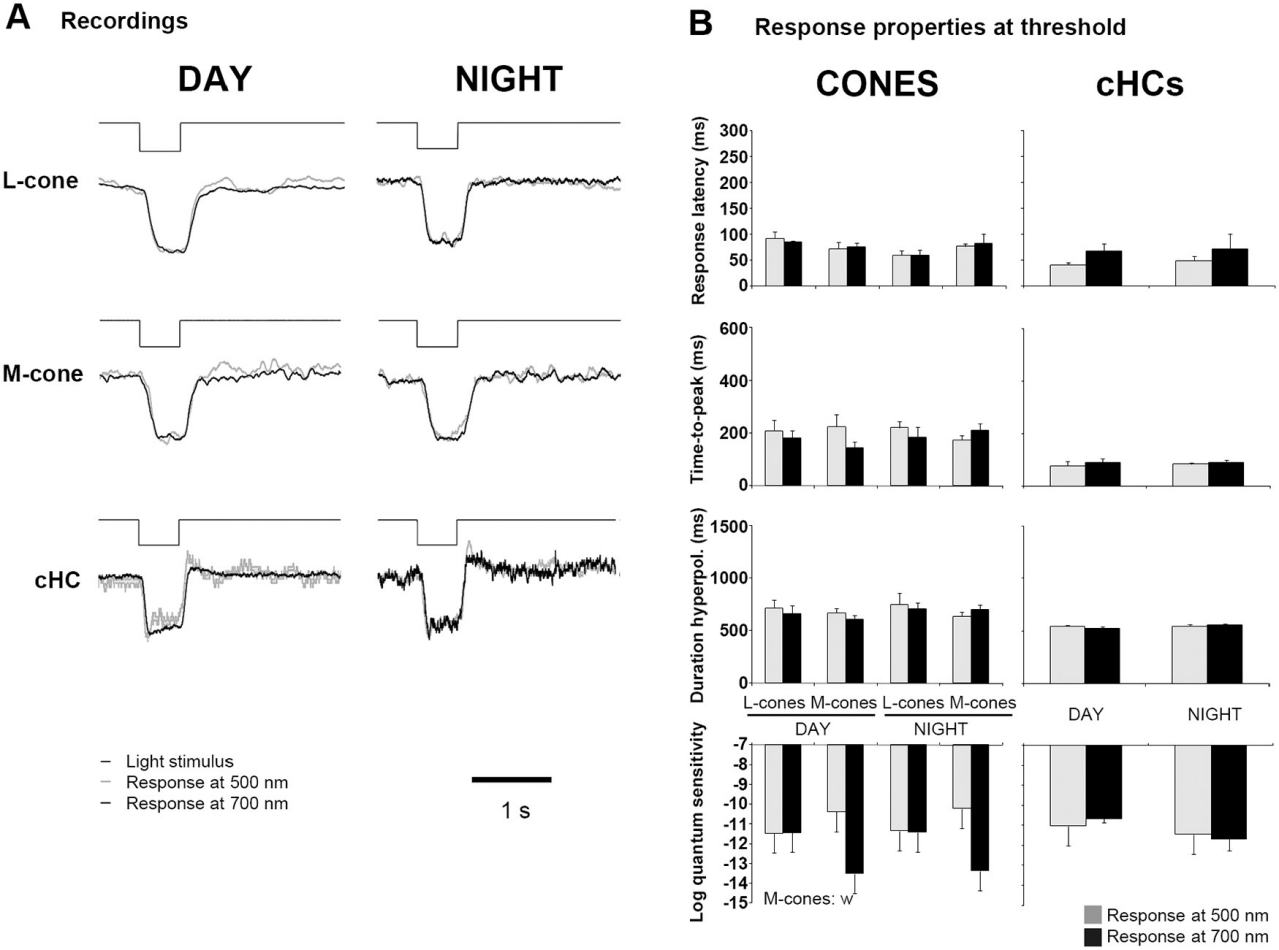
Threshold responses of bright light-adapted cones and cHCs to 500 nm and 700 nm light stimuli during day and night. (A) Representative examples of bright (photopic) light-adapted cone and cHC responses at threshold (0.5 mV) to a light stimulus (500 ms) at 500 nm (grey trace) and 700 nm (black trace) during the day (left column) and night (right column). The amplitude of each trace has been normalized for better comparison. (B) Averaged response latency, time-to-peak, duration of the hyperpolarization, and quantum sensitivity measured in cones (left column) and cHCs (right column) under the conditions described in (A). Data in (B) were analyzed with a two-WAY ANOVA, the first factor was time of day (t) and the second factor was wavelength (w). Significant variations (α = 0.05) are indicated on the left side of each figure.

**Fig 9:**
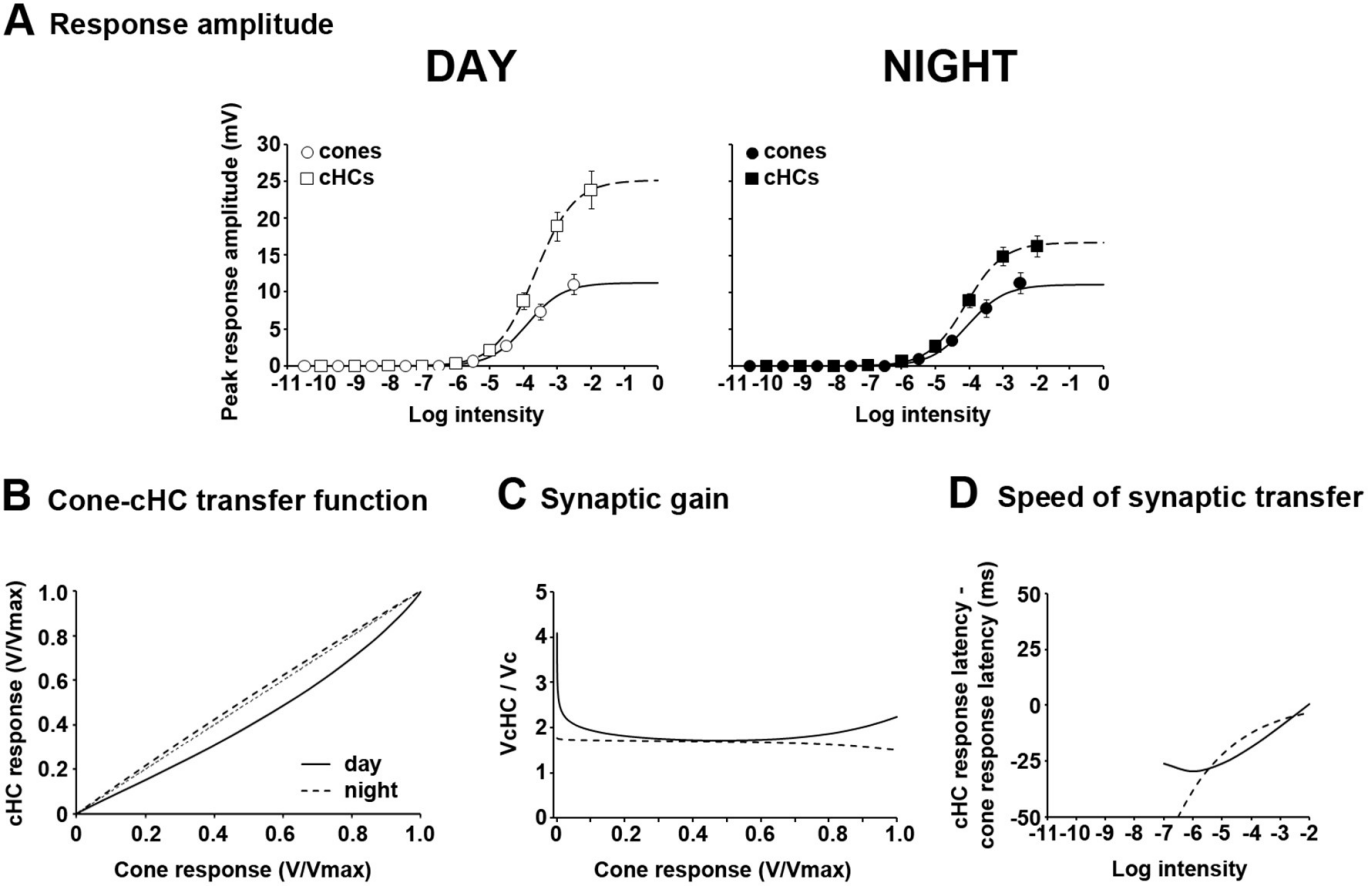
Characteristics of the cone-to-cHC synaptic transfer function during day and night under bright light-adapted conditions. (A) Intensity-response functions of bright (photopic) light-adapted cones (circles) and cHCs (squares) during the day (open symbols; left panel) and night (filled symbols; right panel). Data points were fit to an allometric-type function (see Methods). (B) The cone-cHC transfer function was calculated from the normalized intensity-response functions illustrated in Fig 6B. The result that the transfer function follows the identity line indicates that signal transmission from cones to cHCs is linear. (C) Synaptic gain calculated from the curves illustrated in (A). (D) Speed of synaptic transfer in the day and night was calculated from the curves shown in Fig 7A. Note the similarities between the day and night curves.

The properties of threshold responses to 500 nm and 700 nm-light stimuli resembled those recorded during the day under dark-adapted conditions (compare Fig 8 and Fig 4). The cone-to-cHC transfer function was much more linear during both day and night under light-adapted conditions (Fig 9B) compared to dark-adapted conditions at night (Fig 5B). This linearity is further illustrated by the parallel shapes of the intensity-response functions of cones and cHCs during both day and night under light-adapted conditions (Fig 9A) and the relative invariance of synaptic gain with time of day (Fig 9C). Finally, the speed of synaptic transfer under light-adapted conditions was unaffected by the time of day and always negative, i.e., light responses always developed faster in cHCs than in cones (Fig 9D), as observed during the day under dark-adapted conditions (see Fig 5D). These observations indicate that bright light adaptation overrides the effects of retinal clocks at night on cone light responses and cone-to-cHC synaptic transfer, thus revealing a “negative masking effect” of light on these circadian processes, as has been reported in other non-retina circadian systems [9].

## Discussion

We have previously demonstrated that a circadian clock in the goldfish retina regulates rod-cone electrical and tracer coupling so that coupling is weak during the day and at its maximum at night [19]. As a consequence, rod input, which signals responses to very dim light stimuli, reaches cones and then cHCs at night via open rod-cone gap junctions [19–21]. The aim of this study was to compare the light response properties of cones, cHCs and rHCs to determine the extent to which their circadian changes reflect day/night differences in rod-cone coupling.

The main finding of the present work is that in addition to increasing rod-cone coupling and allowing rod signals to reach cones and then cHCs at night, retinal clocks also modulate cone-to-cHC synaptic transfer. Specifically, we found that the light response latencies of cones and cHCs were increased at night more than that of rHCs (Fig 3). In addition, cone threshold responses to both 500 nm and 700 nm stimuli were slower at night than during the day, an effect that was not observed for rHCs (Fig 4). Moreover, the cone-to-cHC synaptic transfer function became highly non-linear at night with a lower gain than during the day (Fig 5). Consequently, cHC light responses were clipped at night and saturated at a lower intensity (∼ -5.5 log *I*_*o*_) than during the day (∼ -2.5 log *I*_*o*_). Moreover, cone light responses at night saturated at a brighter intensity (∼ -4 log *I*_*o*_) than cHCs (Fig 5). The increase in the non-linearity of cone-to-cHC synaptic transfer at night restricts synaptic signaling to very dim stimuli. This characteristic, together with the increase in rod-cone coupling at night, which enhances the signal/noise ratio of cone light responses and the reliability of cone-to-cHC synaptic transfer [19,35], likely facilitate detection of large dim objects [19,35].

### Circadian clock regulation of cone-to-cHC synaptic transfer

The mechanisms that underlie the change in synaptic transfer at night are unclear but could reflect pre-synaptic, synaptic and/or post-synaptic alterations. These different possibilities are discussed in detail below.

First, day/night changes in rod-cone coupling may contribute to circadian modulation of cone voltage responses and changes of synaptic efficacy. Consistent with an increase in rod-cone coupling at night, we previously reported that the cone input resistance decreases by ~ 3.5-fold at night [19]. Because cones are coupled to rods and to other cones and because rods outnumber cones by 12 to 1 [36], it is possible that cone responses are shunted by neighboring rods at night. It is thus possible that the nocturnal drop in cone input resistance (~ 1 GΩ) shunts the cone response. In addition, because rod-cone gap junctions are between rod spherules and cone pedicles (Fig 1), rod spherule voltage may dominate cone pedicle voltage and transmitter release from cone pedicles at night, and thereby, shape the light responses of cHCs at night. Thus, aspects of cone and cHC light responses can be explained solely by the increase in rod-cone gap junction conductance and the addition of rod input to cones.

Second, it is possible that changes in key membrane properties of photoreceptors and/or cHCs contribute to changes in synaptic gain and kinetics. Two possible important contributors are the membrane potentials and passive membrane properties of the cells. From the data shown in Table 1, we can exclude a significant action of the clock on the resting membrane potential. Indeed, Table 1 shows that the dark resting membrane potentials of cones and cHCs did not change between day and night. We were not able to record the dark resting membrane potential of rods, but we found no difference between day and night for rHCs. Thus, it is unlikely that circadian modulation of the voltage responses of cones and cHCs relies on changes of the dark resting membrane potential of rods, cones or cHCs.

In contrast, passive membrane properties could play a role in the voltage responses of both photoreceptors and their post-synaptic partners. Indeed, our data suggest that the voltage responses of cones are modulated by a circadian clock independently of rod-cone coupling. Because the photo-sensitivities of rods and cones are similar at 700 nm at night (Fig 3D) and because use of full-field light stimuli should minimize shunting of cone light responses by nearby coupled rods, light stimuli at threshold should hyperpolarize rods and cones to a similar extent, which would minimize lateral spread of light-evoked current through the network of coupled photoreceptors. In addition, even if the cone response was shunted at threshold, a fast, transient cone-component should be present at higher intensities. This was not the case as bright light stimuli (> -3 log I_o_) did not produce a cone-like transient response component in cones [19] or in cHCs [20,29] at night. Yet, a cone-like transient response component appeared in cones and cHCs after a few minutes of bright light adaptation [19,20,29] and was clearly present in light-adapted cones and cHCs at night (Fig 8). Thus, the transient-cone component is absent at night under conditions that minimize shunting, but can appear rapidly following light adaptation, suggesting that a circadian clock controls the slow kinetics of the cone response independently of rod-cone coupling.

In addition, the observation that the sensitivity of cones to red light at night is close to that of L-cones during the day (Figs 3D and 4B) confirms that L-cones still respond to light at night, i.e., the intrinsic cone photoresponse is present. However, the means by which retinal clocks could slow down the kinetics of photoresponses at night is still unclear. Changes in photoreceptor membrane resistance likely occur because of daily fluctuations in the extracellular levels of neuromodulators such as adenosine, dopamine, and melatonin, all of which have been shown to modulate membrane conductances by activating transmitter receptors present on photoreceptors [4–8].

It is also possible that the retinal clock controls the kinetics of the phototransduction cascade itself. In fact, key elements and modulators of the cascade are known to be under clock control, including cGMP-gated cation channels [37] and phosducin [38]. Interestingly, a study in larvae zebrafish demonstrated that a retinal clock greatly reduces the a-wave of the electroretinogram, which reflects cone photoreceptor function at night [39]. The idea that the cone photocurrent itself is under clock-control should be studied by recording from cone outer segments with suction pipette electrodes during day and night. Also, it remains to be determined whether cHC membrane resistance changes with the time of day.

Third, the mechanisms that underlie the change in synaptic transfer at night could reflect alterations of key elements of synaptic machinery. For example, the cone pedicle is known to undergo strong morphological changes between day and night. In particular, synaptic ribbons are known to disassemble at night [39–41]. A smaller ribbon would predict that less glutamate is released and/or that the release kinetics are altered. Accordingly, a reduced amount of glutamate in the synaptic space would ensure rapid clearance and clipping of the light response. Thus a day/night change in synaptic ribbon shape could explain the change in cHC response kinetics, including the increase in response latency observed (Fig 5D). Other phenomena may also play a role [8]. For instance, extracellular pH in the retina is under circadian clock control and is lower at night compared to the day, especially in the outer retina [42,43]. Because low pH inhibits cone-to-cHC synaptic transmission in goldfish [42], low pH at night could participate in nocturnal suppression of cone-to-cHC synaptic transmission. In addition, cone L-type voltage-gated calcium channels are known to be under circadian clock-control as well [44].

Finally, the possibility that retinal clocks control the cone-to-cHC transfer function postsynaptically should not be excluded, as circadian clocks have been shown to control postsynaptic glutamate receptor-associated transduction pathways in other brain structures [45]. Yet, it is important to note that key aspects of cHC activity such as the glutamate-gated conductance [46] or the extent of tracer coupling [47] are modulated by dopamine through D_1_ receptors, a type of receptor of low affinity that is insensitive to circadian fluctuations in extracellular dopamine in the retina [47].

Altogether, our observations provide evidence that retinal circadian clocks control cone-cHC synaptic transmission. A variety of clock controlled pre-synaptic, synaptic, and/or post-synaptic mechanisms could explain the day/night shift in synaptic transmission. Additional studies are needed to determine the predominant mechanisms that mediate the actions of the clock.

### Light suppresses circadian clock control of cone and cHC light responses

Following bright (photopic) light adaptation in the day, the light response properties of cones and cHCs resembled those observed during the day following dark adaptation (Figs 6 and 7). That is, following light adaptation at night, the light response properties of cones and cHCs resembled those typically observed during the day under dark-adapted or light-adapted conditions. In all of these experimental conditions, cones receive minimal rod input and cHC light responses are primarily driven by cones. Conversely, at night under dark-adapted conditions, cones receive substantial rod input through open rod-cone gap junctions that connect rod spherules and cone pedicles (Fig 1). Because rods outnumber cones in goldfish retina by 12 to 1 [36], rod spherule voltage dominates cone pedicle voltage and transmitter release from cone pedicles so that cHC light response are dominated by rod input. These observations demonstrate that the nighttime effects of the circadian clock can be fully reversed by bright light adaptation of the retina. This illustrates a “negative masking” effect of bright light at night on clock-driven processes in the retina, a phenomenon that has been observed in many circadian systems [9], including the retina [39].

### Concluding remarks

The data presented here confirm that a circadian clock in the fish retina increases rod-cone gap junction coupling at night in the dark so that rod input reaches cones and then cHCs. As a result, rod voltage dominates cone pedicle voltage and transmitter release from cone pedicles at night. This causes cHC responses to saturate to dimmer stimuli at night than in the day, so that cone-to-cHC synaptic transfer is highly non-linear at night and linear in the day. Our analysis also suggests that the retinal clock decreases the speed of cone-to-cHC synaptic transfer and lowers synaptic gain at night compared to the day. Together, these clock effects tune the cone-to-cHC synapse at night so that it is highly sensitive to small changes in the intensity of very dim stimuli and less responsive to changes in the intensity of brighter stimuli.

## Methods

### Animal tissue/experimental setup

Experiments were conducted on common goldfish (*Carassius auratus*) approximately 15–18 cm in length supplied by Ozark Fisheries, Inc. (Stoutland, MO). This study was carried out in strict accordance with the recommendations in the Guide for the Care and Use of Laboratory Animals of the National Institutes of Health. The protocol was approved by The Ohio State University Institutional Laboratory Animal Care and Use Committee (Protocol Number 2011A00000075-R1-AR2). Goldfish were housed in a 12 hr light/12 hr dark cycle (with lights-on at 3 a.m.) under constant temperature (22° C) for at least 2 weeks before an experiment. Fish were dark adapted for at least 1 hr before surgery. In the case of circadian experiments, fish were dark-adapted for 24–72 hr before surgery. Circadian time was defined by the projected Zeitgeber time from the previous 12 hr light/12 hr dark cycle [19–23]. Goldfish have been chosen for this study because we are able to build on previous work on circadian control of rod-cone coupling in goldfish retina, and because goldfish cones are relatively large in size compared to those found in the retinas of other species, including mammals, facilitating the study of these cells with electrophysiological recording techniques.

Fish were deeply anesthetized with methanesulfanate (MS222, 150 mg.L^-1^), an eye enucleated, and the intact neural retina isolated, as described previously [19–23]. Euthanasia was achieved by decapitation followed by double pithing. All efforts were made to minimize suffering. Isolated intact goldfish neural retinas with photoreceptor side up were superfused in a 2 mL chamber (1 mL/ min) with a bicarbonate-buffered saline solution containing (in mM) 130 NaCl, 20 NaHCO_3_, 2.5 KCl, 10 glucose, 1.0 MgCl_2_, 0.7 CaCl_2_ constantly gassed with a mixture of 5% CO_2_/ 95% O_2_ to maintain pH at 7.5 in the Ringer bottle. The surgery and manipulations of the retina were done using night-vision infrared goggles (Night Optics USA, Inc., Huntington Beach, CA). Experiments were performed under different illumination conditions during the daytime and nighttime of a regular light/dark cycle or during a circadian cycle (i.e. total darkness > 24 hr). All experiments were carried out at room temperature (22° C).

### Patch-clamp recording of goldfish cones

Whole-cell patch-clamp recordings (current-clamp configuration, with I = 0) were obtained from inner segments of goldfish cones, as described previously [19]. Briefly, a custom-made superfusion chamber was placed on the stage of a fixed-stage microscope (BX51WI, Olympus America Inc., Center Valley, PA) in a light-tight Faraday cage. The preparation and microelectrode tips were visualized through a 40X long working distance water-immersion objective (LUMPlanFI/IR, Olympus) under inverted trans-retinal illumination (infrared light > 900 nm, Melles Griot, Rochester, NY). An infrared charge-coupled device (CCD) camera (OLY-150, Olympus) was mounted on the microscope allowing visualization of the preparation on a monitor. Recordings were obtained under visual control with a 3900A amplifier (Dagan Corporation, Minneapolis, MN) using pCLAMP software and digitized with a Digidata 1322A interface (Molecular Devices, Sunnyvale, CA). Signals were filtered at 1 kHz with a four-pole Bessel filter and sampled at 1 kHz. Electrodes were fashioned from borosilicate glass capillaries (OD 1.2 mm, ID 0.69 mm, Sutter Instruments, Novato, CA). The pipette solution contained (in mM) 20 KCl, 100 K-D-gluconate, 7.48 KHCO3, 5.0 HEPES, 1.0 MgCl2, 4.0 Na2-ATP, 0.1 Na3-GTP, and 5 Na2-phosphocreatine. The pH was adjusted to 7.3 with KOH. Biocytin (0.3%) was added fresh daily to a frozen sample of pipette solution. Addition of the tracer lowered the pH to 7.2. Osmolarity was ~ 260 mOsm with biocytin. The tip resistance measured in the bath was ~ 15 MΩ. The seal resistance ranged from 1 to 20 GΩ. Following rupture, the series resistance was 20–30 MΩ.

### Intracellular recording of goldfish cHCs and rHCs

Fine-tipped, intracellular recordings were obtained from L-type (H1) cHCs and rHCs, as described previously [20–23,47–49]. Electrodes were pulled from borosilicate glass capillaries (OD 1.2 mm, ID 0.69 mm, Sutter Instruments, Novato, CA). Electrode tips were filled with 4% biocytin in 5 mM HEPES, pH 7.6, containing 1 M KCl, and then backfilled with 2 M KCl. Final DC resistances ranged between 100 and 200 MΩ. Light exposure of the retinas was minimized by maintaining the preparation in darkness (I < -11 log *I*_*o*_), *i*.*e*., > 4.5 log units lower than daytime cHC threshold. cHCs were impaled without the aid of any light flashes. Cell impalement was then confirmed by briefly flashing a white light (-6 log *I*_o_, 500 ms) at an intensity that does not affect the dark-adapted state of the fish retina in the day or night [19,20,29,35]. Because light stimulus intensities > -4.5 log *I*_*o*_ light adapt the retina and alter the dark-adapted state of the tissue, we studied only 1 cell/retina in dark adaptation experiments. cHCs and rHCs were typically encountered at depths of 80–100 μm and 140–160 μm, respectively, from the outer surface of the photoreceptors. Both cell types had a resting membrane potential of ~ -35 mV in the day and night [20,21,23]. The light responses of the cells were amplified (AxoClamp-2A, Axon Instruments-Molecular Devices, Sunnyvale, CA), visualized on an oscilloscope, sampled at 1 KHz and high-pass filtered at 0.1 Hz. The responses were then digitized (Digidata-1322A, Axon Instruments-Molecular Devices) and sent to a personal computer where they were recorded. Off-line analysis of the light response traces was performed using AxoScope-9 software (Axon Instruments-Molecular Devices).

All recorded cells were identified following injection of the biotinylated tracer biocytin and its subsequent visualization with the avidin-biotin complex and diamino-benzidine reaction (Vector Laboratories, Burlingame, CA) or cyanine 3-conjugated streptavidin (Jackson ImmunoResearch; West Grove, PA), as described previously [19,23,47]. All other compounds were purchased from Sigma (St-Louis, MO).

### Light stimuli

A 100 W tungsten-halogen lamp provided light for a single beam optical bench. Calibrated neutral density filters and narrow-band (± 10 nm) interference filters (from 400 to 700 nm) were used to control light intensity and stimulus wavelength, respectively [19,23,47–49]. Although patch-clamp recording and intracellular recordings were obtained from two different setups with similar light benches, we noticed a slight difference in light intensity at the level of the retina between the two setups: light intensity at the level of the photoreceptors was ∼ 0.5 log unit (∼ 3-fold) dimmer in the patch-clamp recording setup. To compensate for the difference, we subtracted 0.5 log *I*_*o*_ from all cHC and rHC recording data. Thus, data from cone recordings could be compared with cHC and rHC recording data and intensity values indicated in the text are relative to the same unattenuated intensity (*I*_o_), where *I*_o_ = 200 μW.cm^-2^. The maximum, unattenuated light intensity of the stimulus at 500 nm was 1.67x10^13^ photons.cm^-2^.s^-1^, which was estimated to equal 1.49.10^5^ R*.rod^-1^.s^-1^. The peak sensitivity (*k*; see below) was converted to mean isomerization rate per rod according to the expression:

$${Rh}^{*}.{rod}^{-1}.s^{-1}=\frac{{\left[{antilog}_{10}(k)\right]}^{-1}\;.\;Q_{eff}Ab\;.\;Q_{eff}Iso}{d_{rod}}$$

with quantum efficiency of absorption (Q_eff_Ab) = 20%; quantum efficiency of isomerization (Q_eff_Iso) = 67%; and the density of rods (d_rod_) = 15.10^6^.cm^-2^. When required, light adaptation was achieved by repetitively flashing a bright photopic light (500 ms, -2/-3 log *I*_o_ flashes at 0.125 Hz) for more than 60 min.

The term “scotopic” refers to the range of very dim ambient illumination that typically occurs during a moonless night to which rods, but not cones, which have been separated from the retina, can respond. Conversely, the term “photopic” refers to the range of bright ambient illumination that typically occurs on a sunny day to which cones, but not rods, can respond. Lastly, the term “mesopic” refers to the range of ambient illumination between scotopic and photopic, which typically occurs at dusk and dawn, to which both rods and cones can respond.

### Data analysis

We used the following definitions to quantify the light response kinetics of cones, cHCs and rHCs:

*A hyperpolarizing light response* was defined as a downward deflection of the membrane potential equal to or greater than two times the amplitude of the noise; the *response latency* was defined as the time between light stimulus onset and the beginning of the hyperpolarizing response; the *response amplitude* was the difference between the resting membrane potential and the membrane potential at the peak of the response; the *time to peak* was defined as the time between the beginning of the light response and its maximum amplitude (i.e., the most negative value of the membrane potential); and the *duration of the response* was the duration of the hyperpolarization. Indeed, the appearance of a depolarizing component at the end of the light response was not consistent from cell to cell and was not analyzed further. The end of the response was thus set as the time the membrane potential equaled the initial resting potential for the first time following the light-evoked hyperpolarization. For spectral sensitivity measurements, a 0.5 mV criterion response was used. Relative sensitivity was expressed as the log of the ratio of quantum sensitivities measured at 500 and 700 nm at threshold (RS = log [S_500_/S_700_]). All data are presented as mean ± SEM unless otherwise noted. Differences between two groups were tested using the Student’s t-test and between more than two groups using the Tukey post hoc test, following one-WAY or two-WAY ANOVA.

### Regression models

Non-linear least-squares regression analysis of the data was performed using Origin 7.0 (OriginLab, Northampton, MA).

The absolute values or the normalized (against the maximal amplitude) values of the peak amplitude plotted against light intensity were fit to a hyperbolic Hill-type function in the form:

$$V=Vmax\;.\;\frac{I^{n}}{\left(I^{n}+\;K^{n}\right)}$$

where *V* is the response amplitude, *V*_*max*_ is the maximum response amplitude, *I* is the intensity, and *K* the semi-saturating constant (intensity to generate half-maximum amplitude). The Hill coefficient n defines the steepness of the intensity-response curve.

Latency, time-to-peak, and duration were plotted against light intensity. Data points were fit to an allometric-type function in the form:

$$L=a\;.\;I^{b}$$

where *L* is the response latency, time-to-peak, or duration, *a* is the allometric constant, *I* is the intensity, and *b* the allometric exponent.

Spectral sensitivity data were corrected for equal energy and expressed as Log Quantum Sensitivity or normalized at the wavelength of peak sensitivity (Log Relative Quantum Sensitivity), averaged and plotted against the wavelength of the light stimulus (λ). Statistical analysis of cone spectral sensitivity was done using nonlinear least squares regression of our experimental data with the published template for goldfish visual pigments [28]. Nomograms were generated from the template with λ_max_ = 516 nm (goldfish rod porphyropsin), 451 nm (goldfish blue cone pigment, S), 539 nm (goldfish green cone pigment, M), and 608 nm (goldfish red cone pigment, L) [28]. The modified nomogram under dark-adapted conditions at night was calculated by combining the rod and L cone nomograms weighted by their relative difference in sensitivity at λ_max_ [19].

The cone-cHC transfer function, the voltage gain function, and the speed of synaptic transfer were calculated from the regression curves.
